# Phylogenetic Analysis of *Theileria annulata* Infected Cell Line S15 Iran Vaccine Strain

**Published:** 2012

**Authors:** GH Habibi

**Affiliations:** Department of Parasitology, Vaccine Research and Production, Razi Vaccine and Serum Research Institute, Karaj, Iran

**Keywords:** *Theileria*, Vaccine strain, Phylogenetic Analysis, PCR, 18S rRNA gene

## Abstract

**Background:**

Bovine theileriosis results from infection with obligate intracellular protozoa of the genus *Theileria*. The phylogenetic relationships between two isolates of *Theileria annulata*, and 36 *Theileria* spp., as well as 6 outgroup including *Babesia* spp. and coccidian protozoa were analyzed using the 18S rRNA gene sequence.

**Methods:**

The target DNA segment was amplified by PCR. The PCR product was used for direct sequencing. The length of the 18S rRNA gene of all *Theileria* spp. involved in this study was around 1,400 bp.

**Results:**

A phylogenetic tree was inferred based on the 18S rRNA gene sequence of the Iran and Iraq isolates, and other species of *Theileria* available in GenBank. In the constructed tree, *Theileria annulata* (Iran vaccine strain) was closely related to other *T*. *annulata* from Europe, Asia, as well as *T. lestoquardi*, *T. parva* and *T. taurotragi* all in one clade.

**Conclusion:**

Phylogenetic analyses based on small subunit ribosomal RNA gene suggested that the percent identity of the sequence of Iran vaccine strain was completely the same as Iraq sequence (100% identical), but the similarity of Iran vaccine strain with other *T. annulata* reported from China, Spain and Italy determined the 97.9 to 99.9% identity.

## Introduction

Bovine theileriosis results from infection with obligate intracellular protozoa of the genus *Theileria*. The two most important species in cattle and water buffalo are *T*. *parva*, as the agent for causes east coast fever, and *T*. *annulata*, which causes tropical theileriosis. *Theileria* *parva* occurs in 13 countries in sub-Saharan Africa causing East Coast fever (ECF), Corridor disease, and January disease. *Theileria annulata*, the cause of tropical theileriosis, occurs in large parts of the Mediterranean coast of north Africa, extending to northern Sudan, and southern Europe. South-eastern Europe, the near and Middle East, India, China and central Asia are also affected (1–3).

*Theileria* is classified based on microscopic observations, ultrastructural features, lifecycle, geographic region and vertebrate and non-vertebrate host (4). Clinical classification categorizes *Theileria* to malignant, moderate and benign species. Nonetheless, the exact taxonomic *Theileria* spp. have been difficult to establish and the subject of considerable debate (5–7). There is a number of factors makes complexity of assigning taxonomic positions, including similar morphology among this group of parasites regardless of vertebrate host, incomplete lifecycle data, serologic tests that are not specific enough to discriminate individual species in the presence of mixed infections, and the difficulty of obtaining pure isolates for studies when the circulating parasitemia may be very low (8–10).

The advances in molecular biology and specially sequence data analysis allowed the researchers to identify and characterize the hemoparasites species in particular *Theileria* group. Ribosomal RNA is the most abundant constituent of nucleic acids in any non-viral organism with the eukaryotic RNA transcription unit consisting of the large and small subunit (18S rRNA) and the 5.8S rRNA gene (11). The 18S rRNA gene is increasingly accepted as a widely used marker for characterization, taxonomic classification, and phylogenetic analysis and this gene has been sequenced from a variety of different organisms, resulting in a large database for sequence comparisons (6, 10, 12–22). The conserved function and structure of the 18S rRNA molecule allow sequences to be aligned, even among divergent species. However, the molecule also possesses phylogenetically informative variable regions that are useful for determining relationships among species (23).

In this study, we decided to determine the phylogenetic position of *T. annulata* infected cell line S15 Iran vaccine strain and *T*. *annulata* Iraq field isolate with 36 different *Theileria* spp. and six out-group protozoan parasites using 18S rRNA gene sequences comparison.

## Materials and Methods

### Parasites

Two *T. annulata* strain/isolates were used in this study. *Theileria* *annulata* infected cell line (S15 Iran vaccine strain, Tehran, Vasfenard) and Iraq *T.annulata* field isolate were provided from Protozoology and Vaccine Production Department of Razi Institute and Duhok Province, Kurdistan of Iraq respectively.

### DNA isolation

Proteinase K and further phenol chloroform purification were performed for DNA extraction (24). Briefly, after treating the cells with lyses buffer, followed by centrifugation, proteinase K and SDS solution was added to the pellet, and then was incubated until most of the cellular protein was degraded. The digest was deproteinized by phenol/chloroform/isoamyl alcohol extraction, recovered by ethanol precipitation, then was dried and resolved in TE buffer. DNA concentration was determined either by agarose gel electrophoresis and spectrophotometry (A260) and measuring the ratio of A_260_/A_280_. Moreover, quality of the isolated DNA was evaluated by agarose gel electrophoresis.

### PCR Primer Design

The specific primers were designed based on *T. annulata* 18S ribosomal RNA gene sequence (accession # EU083801) (by CinnaGen, Iran). Two primer pairs were designed in order to span the major hyper variable regions along the 18S ribosomal RNA gene sequence (Gene Runner program, Version 3.05). The first two primers, F1 (5’ GGC GGC GTT TAT TAG ACC 3’) and R1 (5’ TCA ATT CCT TTA AGT TTC AGC C 3’) were used to amplify bases between 186-1093 and the second primers, F2 (5'CAG ATA CCG TCG TAG TCC 3’) and R2 (5’ CCT TGT TAC GAC TTC TCC 3’) were applied to amplify bases between 945-1714 of *T. annulata* 18S ribosomal RNA gene sequence (EU083801) and these two primers sets covered the majority length of 18S ribosomal RNA gene sequence with 127 bp overlapping.

### Polymerase Chain Reaction

PCR was performed in a final reaction volume of 20 µl containing 50 mM KCl, 10 mM Tris-HCl (pH 8.3), 1.5 mM MgCl_2_, 0.1% Triton X-100, 200 µM (each) deoxynucleoside triphosphate, 0.5 U of *Taq* DNA polymerase (CinnaGen, Iran), 10 pmol of each primers, and 2 µl of DNA template. The reactions were performed in an automatic DNA thermal cycler (Techne, Germany) with the first incubation at 94 ^o^C for 3 min and were pursued by 35 cycles. Each cycle consisted of a denaturing step of 20 seconds at 95 ^o^C, an annealing step of 45 seconds at 56 ^o^C, and an extension step of 50 seconds at 72 ^o^C, followed by final extension step of 10 min at 72 ^o^C.

### PCR product detection and sequencing

Amplified PCR products were separated by electrophoresis on a 2% agarose gel, stained with ethidium bromide, and visualized by UV transillumination. PCR products were cleaned and extracted from agarose gel and were submitted for bidirectional DNA sequencing by using chain termination method (MWG, Germany). The provided sequences from F1R1 and F2R2, first were merged and offered to be aligned for multiple sequence alignment and phylogenetic study of *Theileria* spp. and outgroups as well.

### Sequence alignment and phylogenetic analysis

The DNA sequences of 18S rRNA gene obtained from two studied *T. annulata* samples and 42 sequences of 18S rRNA gene sequences including 36 *Theileria* spp., 4 *Babesia* spp. and two coccidian protozoa were accessed from GenBank. The sequences were aligned by Clustal W multiple alignments program (25). The alignment was manually edited in BioEdit and truncated to the size of the smallest sequence (∼1,400bp). Phylogenetic tree was constructed by using DNADist Neighbor-Joining method (version 3.6a2.1), sequence identity matrix of all sequences were computed as well (BioEdit phylogeny package, Version 7.0.1).

### Nucleotide sequence accession numbers

The 18S rRNA gene sequences of the *T. annulata* S15 Iran vaccine strain and *T. annulata* field isolate from Iraq, Duhok have been submitted to GenBank and can be retrieved under accession numbers of HM628581, and HM628582 respectively. All 42 small subunit ribosomal RNA gene sequences were used for this phylogenetic study were listed in Table 1.

**Table 1:** The sequence identity values between *T. annulata* Iran S15 Vaccine Strain and 43 small subunit of Ribosomal RNA gene sequences belong to different *Theileria* species and four *Babesia* spp. as well as two outgroup coccidians, *Toxoplasma* and *Isospora*. Different *Theileria* species are categorized in six clades (Q, Y, O, M, B and A) in addition to two outgroup clades for *Babesia spp*. and Coccidian spp.

Sequence	Accession number	Clade *	Identity percent **
***T.equi***	ab515315		90.1
***T.equi***	ay534882	**Q**	91.5
***T.equi***	eu888906		88.2
***T.youngi***	af245279		91.1
***T.bicornis***	af499604	**Y**	92.7
***T.velifera***	af097993		95.3
***T.capreoli***	ay726011		96.4
***T.ovis***	ay260171		97.3
***T.ovis***	ay260172		97.3
***T.ovis***	eu622911	**O**	97.1
***T.ovis***	ay533144		97.0
***T.ovis***	fj603460		97.4
***T.mutans***	af078815		94.2
***T.mutans***	fj213586	**M**	92.9
***T.buffeli***	dq287959		95.7
***T.buffeli***	ef126184		96.1
***T.buffeli***	af236094		95.9
***T.buffeli***	dq104611		95.9
***T.buffeli***	z15106		95.9
***T.buffeli***	af236097	**B**	95.9
***T.buffeli***	fj426360		96.6
***T.sergenti***	gu143088		92.1
***T.sergenti***	fj225392		95.5
***T.sergenti***	eu083803		95.8
***T.separata***	ay260175		95.4
***T.orientalis***	ab520958		95.9
***T.sinensis***	eu274472		96.0
***T.parva***	l02366		98.5
***T.taurotragi***	l19082		97.9
***T.annulata***	eu073963		99.8
***T.annulata***	eu083799		99.4
***T.annulata***	eu083800		99.8
***T.annulata***	eu083801	**A**	99.9
***T.annulata***	fj426369		99.2
***T.annulata***	dq287944		99.6
***T.annulata*** **, Iran Vaccine Strain**	hm628581		id
***T.annulata*** **Iraq**	hm628582		100.0
***T.lestoquardi***	AF081135		99.5
***B.ovis***	ay150058		85.3
***B.motasi***	ay533147	***Babesia***	87.0
***B.divergens***	AY572456		88.7
***B.cabali***	AY534883		87.6
***Toxoplasma gondii***	L37415	**Coccidian**	84.4
***Isospora suis***	U97523		83.3

* The analyzed sequences were grouped in eight major clades, based on 18S rRNA gene sequences

** Nucleotide identities are given in percentage

## Results

**Fig. 1:**
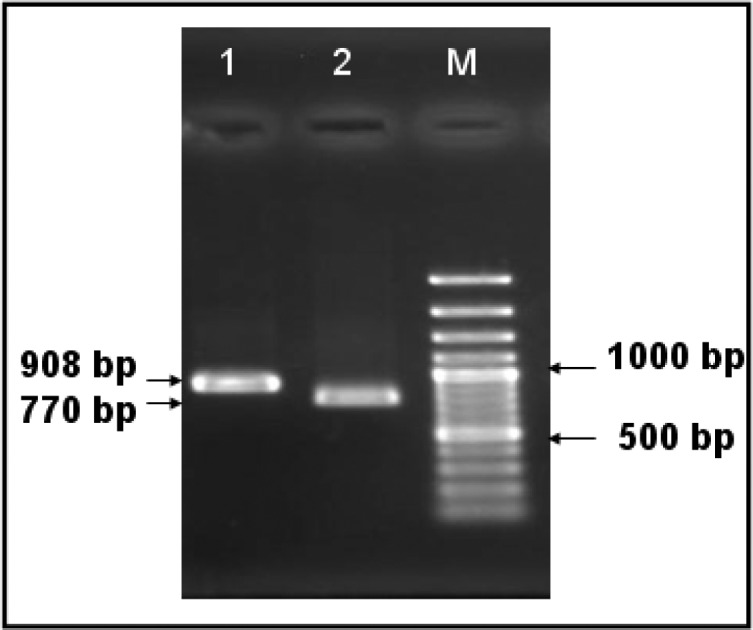
Gel agarose (2%) electrophoresis of amplified 18S rRNA gene sequence of *Theileria annulata*. Two fragments of 18S rRNA gene sequences were amplified for *Theileria annulata*. Lane 1; fragment of 908 bp, lane 2; fragment of 770bp and, M; 100 bp DNA ladder as size marker

The expected amplicons with sizes of 770 and 908 base pairs (bp) were observed in all of the examined samples (Fig. 1). The sequences of the 18S rRNA gene of the *T. annulata* Iran vaccine strain and Iraq (Duhok isolate) were determined from the overlapping flanking sequences of two generated PCR fragments. The sequencing of the PCR products yielded 1424 and 1413 base pair length for two Iran and Iraq samples respectively. The sequences were then subjected to phylogenetic analysis by using the BioEdit programme. The sequence identity matrix was also determined and showed 88.2-100% homology between *T. annulata* 18S rRNA sequence of S15 Iran vaccine strain and *Theileria* spp. from all over the world (Table 1). The sequence of Iran vaccine strain showed completely same as Iraq sequence (100% identical). But the similarity of Iran vaccine strain with other *T. annulata* reported 18S rRNA gene sequences from China, Spain and Italy determined the 97.9 to 99.9% identity. The closest similarity between *T. annulata* Iran vaccine strain and other *Theileria* species was belonged to *T. lestoquardi* Iran vaccine strain (AF081135) 99.5% similarity. There were selected 6 outgroup 18S rRNA gene sequences from *Babesia ovis*, *B. motasi*, *B. cabali*, *B. divergens*, *Toxoplasma gondii* and *Isospora suis* those exhibited the most difference among the comparison (88.3-88.7% similarity).

### Entropy plot

**Fig. 2:**
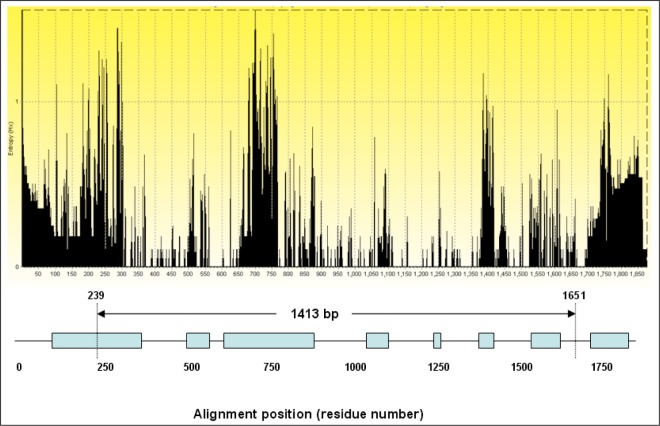
Plotted entropy shows the 4 major hypervariable regions along the 18S rRNA gene sequence. Two studied *T.annulata* sequences had 1413bp length. The plot shows the fragment of *Theileria annulata* have an enough length to cover the majority of hypervariable regions. The boxes are the hypervariable regions along the gene sequence and the arrows demonstrate the region was sequenced and applied for phylogenetic analysis

The alignment was manually edited in BioEdit software and truncated to the size of the smallest sequence (1,413 bp). After resizing the aligned 44 sequences, in order to have the correct comparison, the designed entropy plot was plotted and this design showed the amplified 1413 bp length of the 18S rRNA gene sequence in this study, spans most of the hypervariable regions exist along the alignment (Fig. 2).

### Sequence alignment and phylogenetic analysis

The phylogenetic tree was constructed based on the *T. annulata* Iran S15 vaccine strain, Iraq isolate, 36 *Theileria* spp. and 6 out-group sequences including *Babesia* spp., *Toxoplasma gondii* and *Isospora suis* sequences (Table 1).

**Fig. 3:**
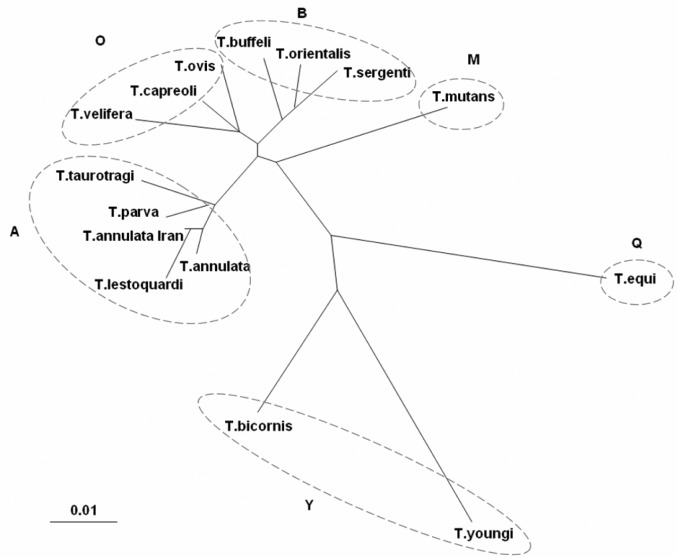
Unrooted phylogenetic tree is constructed using *Theileria* spp. 18S rRNA gene sequences. *Theileria* spp. sequences were grouped in six major clades (detailed description is in the text). Scale bar represents nucleotide substitutions per position

*Theileria* sequences was divided into six clades in the constructed tree, including “Q” clade; consists *T*. *equi*, “Y” clade; includes *T*. *youngi* and *T. bicornis*, “O” clade; contains *T. ovis*, *T*. *capreoli* and *T*. *velifera*, “M” clade; includes *T. mutans*, “B” clade; consists *T*. *buffeli*, *T*. *sergenti*, *T*. *sinensis*, *T*.*separata* and *T*. *orientalis*, “A” clade; includes *T. annulata*, *T*. *parva*, *T*. *lestoquardi* and *T. taurotragi*, and two more clades for outgroup sequences; “*Babesia*” clade; consists *B. ovis*, *B. divergens*, *B. motasi* and *B. cabali*, and “Coccidian” clade; contains *Toxoplasma gondii* and *Isospora suis* (Table 1 and Fig. 3).

There are eight *T. annulata* 18S rRNA gene sequences in clade “A”, identity percent of *T. annulata* Iran vaccine strain and other *T. annulata* in this clade are 99.2% to 100%.

The identity percent between *T. annulata* Iran strain and *T*. *lestoquardi* was 99.5% and this similarity rate was more than the identity percent between *T. annulata* Iran and Italy or China, Xinjiang isolates.

## Discussion

Based on available literature, this is probably the first phylogenetic analysis, molecular characterization of *T. annulata* infected cell line of Iran vaccine strain, and a field isolate from clinically infected cattle in Iraq, Duhok by using 18S rRNA gene sequence.

One of the most important points in Good Manufacturing Practice (GMP) is to characterize the local vaccine seed in Razi Vaccine and Serum Research Institute, Karaj, Iran. Therefore, we decided to classify the *T. annulata* Iran vaccine strain cell line. Hence, we focused on to establish the phylogenetic relationships of Iran *T. annulata* vaccine strain with other *Theileria* species using 18S rRNA gene sequences.

Basically, there are three steps in all phylogenetic analysis; multiple alignment of the sequences, distance calculation and tree construction. Using taxa (the outgroup) that are known to fall outside of the group of interest (the ingroup) is the way to root tree. In this study, we applied *Babesia* species and two coccidian parasites to root *Theileria* species.

**Fig. 4:**
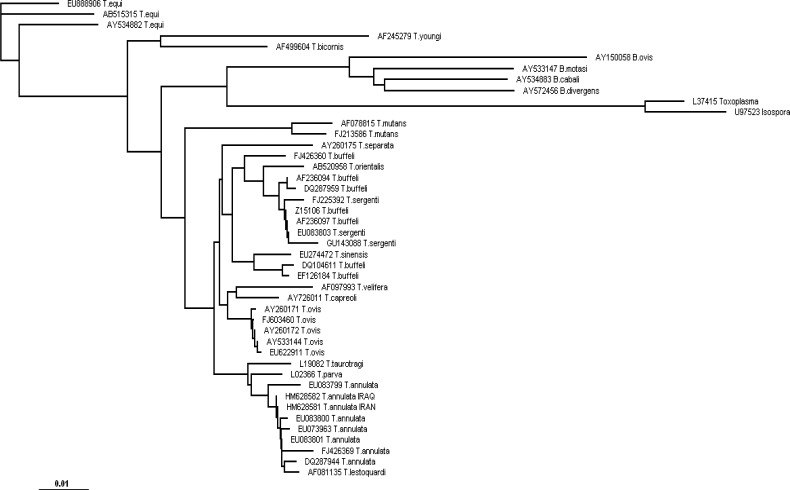
Rooted phylogenetic tree of gene sequences of *Theileria annulata* S15 Iran vaccine strain, Iraq isolate and other *Theileria spp*. also outgroup 18S rRNA gene sequences existing in GenBank (Outgroup sequences are the least related to the group of taxa that we are studying). The outgroups are considered for rooting a tree. Scale bar represents nucleotide substitutions per position

In addition, *Theileria* species are host and vector specific (26, 27) but in some countries more than one species can infect animals, which causes a problem in diagnosis and epidemiology. Although recent molecular studies suggest that the genus *Theileria* including *T*. *annulata*, *T*. *parva* and *T*. *lestoquardi* are very similar in features of microscopic characteristics; these species are phylogenetically distinct and can be differentiated by accurate molecular techniques (28, 29). Although, *Theileria* spp. are tick-transmitted and the parasite is fully corresponded to the specific hosts, and this association makes a geographical distribution of the *Theileria* species around the world, but the phylogenetic analysis clearly shows close relationship of different species, *T. annulata*, *T. lestoquardi*, *T. parva* and *T. taurotragi* in constructed tree all are within clade “A” (Fig. 4).

In this study, two fragments of 18S rRNA gene sequences of *T. annulata* 770bp and 908bp were amplified for two examined samples. Nucleotide sequence identity data demonstrated that the Iran S15 vaccine strain (GenBank Accession Nr: HM628581) has nucleotide homology of 99.9% with China, Neimeng (EU083801), 99.8% with China, Ningxia (EU083800), 99.8% with China, Yining (EU073963), 99.2% with Italy (FJ426369), 99.6% with Spain (DQ287944), 99.4% with China, Xinjiang (EU083799) and 100% with Iraq, Duhok isolate (HM628582). From this perspective, the S15 live attenuated vaccine strain has a great homology with other *T. annulata* isolates; Iraq in neighborhood and China, Spain and Italy far from Iran. But the effectiveness of this vaccine for use in these countries as a valuable vaccine is an objective of future studies.

The minimum identity belongs to *T. annulata* Italy isolate and maximum similarity was for Iraq isolate. The interesting finding is that all pathogenic *Theileria* species including *T. annulata*, *T*. *parva* and *T*. *lestoquardi* are in “A” clade (Table 1).

Furthermore, *T*. *lestoquardi* and *T*. *annulata* share an extremely high similarity (99.5%) in their 18S rRNA gene sequences. This finding and the recent occurrence of *T*. *lestoquardi* infection in bovine cells might be a good explanation for very high closeness of these two *Theileria* species.

In the present study, another sequence identity analysis was performed by using a smaller size of 18S rRNA gene sequence of 793 nucleotides. The *T*. *annulata* field isolate from Golestan Province of Iran (HM535613) showed the 100% homology with the *T*. *annulata* Iran S15 vaccine strain, Iraq Duhok and Turkey isolates (data not shown).

Molecular phylogeny was used to classify *Theileria* spp. obtained in the Hubei province of China; the results clearly determined that the *Theileria* *spp*. from ruminants found in Hubei belonged to the benign group of *Theileria* spp. (30).

Three *Theileria* genotypes were phylogenetically analyzed by Nagore et al. in Spain that, sharing 96.7%–97.0% similarity between their 18S rRNA gene sequences: *T*. *ovis*, *Theileria* *sp*. OT1, and *Theileria* sp. OT3 (29).

Yin et al. in China suggested that the infective *Theileria* for small ruminants are comprised of two species with the same morphology, vector and life cycle. Regarding to the classical taxonomy, they should be one species, but the phylogenetic tree showed several *Theileria* spp. including China1 and 2 that were isolated from sheep and goats (31).

Nijhof et al. showed the cause of deaths in wild ruminants in South Africa that was previously attributed to the genus Cytauxzoon, but phylogenetic analysis has clarified the *Theileria* is the cause of mortality (32).

In conclusion, according to the data presented here, there are high homologies between *T. annulata* Iran 18S rRNA gene sequence strain with other *T. annulata* from above mentioned countries, in particular Iraq and Turkey. Therefore, if this phylogenetic data correlate with immunological response of susceptible cattle, it might be a new sight to find an efficient vaccine to control and prevention of Tropical Theileriosis through molecular epidemiological methods.
